# Progress in Skin Diseases

**Published:** 1901-05-25

**Authors:** 


### Progress in Skin Diseases.

(*Continued from page* 119.)

**Erythema bullosum.** — Graham Chambers [16] points out the multiformity of the lesions, the presence of one or more of the iris forms and the predilection of the disease for the face, hands, legs, and mucous membrane of the mouth. On the other hand, pemphigus is usually chronic, the lesions less symmetrical, and rarely concentrically arranged as is the case in E. bullosum. T. Sydney Short [17] reports a very severe case coming on suddenly in a healthy woman. The whole of the buttocks, hips, and portions of the thighs were completely flayed, and large areas over the chest and legs were blistered and the skin left hanging to them in places. Around some of the blebs there was a wide area of erythema, while others arose from healthy skin. She had taken a meal of fish and pork the day before the rash came out. There was no burning, itching or other irritation, but pyrexia and some delirium at night. Some of the patches showed a bluish centre surrounded by a white ring and that by a red one. The lesions healed rapidly, and in about a month she was quite well. The diagnosis was made from D. herpetiformis and general eczema, chiefly by the absence of sensory symptoms. Pemphigus would cause more severe constitutional symptoms, and recovery would be slow if the patient survived. The amount of basal erythema in pemphigus is very small.

**Lupus Erythematosus.**—Galloway [18] showed a case where not only the face and hands, but even the palate was affected. The affection of the mucous membranes in chronic cases seems to be very rare. Abraham finds that an application of carbolic and camphor, with quinine internally, is very serviceable. The syphilitic forms are numerous, and some, according to Jonathan Hutchinson, [19] do not ulcerate. He discusses a patient in whom the eruption had travelled over the whole of the face, and fresh patches had developed on the old ground. Extensive areas on the back, symmetrical lesions of the feet, clefts between the toes and sclerosis of the tongue helped to show the specific nature. There were no apple jelly nodules, but tubercles slightly ulcerated with abrupt margins instead of with a brown infiltration of the skin. Kreibich [20] reports the occurrence of multiple carcinoma in a case of L. erythematosus. The inflammatory condition probably rendered possible the abnormal proliferation of the epithelium.

**Alopecia.**—R. Ryan [21] showed a case rapidly cured with lysol. He commenced with a 10 per cent. solution and increased it to 40 per cent. Some persons could not bear 5 per cent. O. Lassar [22] in discussing the causation shows that the tropho-neurotic theory is very improbable, but there is evidence of contagiousness, though the organism has not been discovered. He washes the scalp daily with a strong tar soap, and, after drying, applies successively a 2 per cent. sublimate solution, then absolute alcohol with ½ to 1 per cent. naphthol, and finally 2 per cent. salicylic acid in oil. He thinks the Finsen rays may eventually be valuable. Graham Little [23] showed a case of nearly universal alopecia following measles in a girl of 4 years. Rapid recovery took place under treatment with pilocarpin ointment, 4 grains to the ounce, applied twice a day. Scheffer [24] injects under the scalp all around the bare patch a solution containing corrosive sublimate and pilocarpin. The former destroys infecting organisms, and the latter stimulates the hair follicles which are related to sweat glands and like them easily affected by pilocarpin. He claims that very satisfactory results follows, some new growth being generally seen in a fortnight. Jacquet [25] disbelieves in the microbic origin, since there is no evidence of inoculability. The bulbs are destroyed long before the hair falls out by a form of atony affecting the vessels and the connective tissue. He recommends frequent massage with friction and percussion. Sabourand [26] adheres to the view that there are two varieties of the disease, the one, *pelade seborrhéique*, due to micro-organisms, which is contagious, and the other *pelade ophiasique*, which is probably hereditary and non-contagious. R. Leftwich [27] recommends for *seborrhea* the application of benzene, perfumed with oleum geranii (♏x to the ounce if desired). This is painted on once a week, and it immediately removes the crusts. In mild cases equal parts of spirit and benzene may be used instead. As the benzene leaves the scalp very dry, castor oil with bay rum and cantharides may be applied every morning.

**Psoriasis.**—Abraham [28] has found no treatment for psoriasis of the scalp equal to an ointment containing one drachm of ammoniate of mercury with three and a half drachms of soft soap and vaseline well rubbed in every night. The most inveterate attacks disappear under this treatment in a few weeks, and sometimes do not recur for long periods. Probably the earliest instance of an attack of psoriasis is one recorded by J. H. Rille[29], when the patient was only five days old. It may follow eruptive fevers or vaccination, and outbreaks have been reported at three, four, and eight months.

**Epidermolysis bullosa.**—This affection would formerly have been classed under pemphigus, but of late much attention has been directed to its peculiarities. Bullæ and vesicles tend to appear after any stimulus or slight injury. There is at first a narrow erythematous margin, but this soon fades, and if the vesicle is not broken it dries up in ten days, and leaves a scale but no scar. The frequency of the attacks decreases from childhood to old age, and is greatest in males and during hot weather. It affects several members of a family, and in severe cases there is so much destruction of the skin that any active occupation is impossible. As Elliott[30] pointed out the lower layers of the retemucosa are kept bathed in an exudation which leads to degeneration of the epithelium and the formation of bullæ.

**Urticaria pigmentosa.**—A case from South Africa showed numerous reddish-brown maculæ, not definitely grouped, the recent ones being slightly raised. There had been much pruritus. In another patient from South Africa, shown by S. Mackenzie,[31] the lesions followed the more usual type, but the general appearance was very similar. Galloway[32] notices that the lesions are composed of an infiltration of the tissues with "mast" cells. In a male infant the reddish-brown papules and macules began to appear in the course of an attack of varicella, while true urticaria will in some persons follow any contact with the Rhus toxicodendron, phenyl-hydrazin, formalin, and other irritants.

**Acne hypertrophica.**—Romme[33] excised the separate masses and treated the raw surfaces with the thermo cautery, and when they granulated reduced them with nitrate of silver. For lighter cases he advises electrolysis with a current of 1 to 5 milliamperes, the needle being inserted into each sebaceous opening and along each varicose vein.

**Acne Rosacea.**—Weak solutions of ichthyol externally with 15 to 30 grain doses daily given by the mouth, are praised by Alex Brownlie.[34] He steams the face night and morning, then washes it with ichthyol soap, and afterwards in plain water. If it can be borne, an ointment of the same with or without white precipitate, is applied after the washing. He employs in Acne vulgaris strong ichthyol and sulphur soap, with brisk rubbing by a flesh glove. In the pustular form H. Leslie Jones[35] advises perforating each pustule by a finely pointed stick dipped in nitric acid and turned rapidly to destroy the walls. Several pustules may be done at each sitting. He paints the face with turpentine twice or more daily, but insists on the care of details needed to prevent scars in the little operation, and needless pain.

W. J. Munro[36] recommends supra-renal extract in A. rosacea. After steaming the face in the morning he orders a calamine and sulphur lotion, and at night the patient paints it with a soloid of the extract dissolved in a drachm of water. This causes a temporary smarting and redness, which is followed by anæmia. Besides correcting dyspepsia and other troubles, he gives from one to six tabloids of supra-renal internally every day, and the results are said to be most satisfactory.

16 Brit. Jour. Derm., Jan., p. 38. 17 Birm. Med. Rev., Feb. 18 Brit. Jour. Derm., Jan., p. 7. 19 Clinic Jour., Dec. 5. 20 Amer. Jour. Med. Sc., Jan. 21 Brit. Jour. Derm., Jan., p. 27. 22 Do., Jan., p. 36. 23 Do., Jan., p. 18. 24 Brit. Med. Jour., Dec. 8. 25 Edin. Med. Jour., Jan. 26 Practit. Nov. 27 Brit. Med. Jour., Jan. 5. 28 Lancet, Sept. 22. 29 Amer. Jour. Med Sc. 30 Practit., Nov. 31 Brit. Jour. Derm., Jan., p. 19. 32 Practit., Nov. 33 Brit. Med. Jour., Dec. 8. 34 Lancet, Nov. 24. 35 Brit. Med. Jour., 1901, p. 513. 36 Austral. Med. Gaz., Dec. 20.

